# Post-COVID-19 Thyroid Dysfunction and Autoimmune Ultrasound Changes: A 12-Month Prospective Cohort Study

**DOI:** 10.3390/medicina62071354

**Published:** 2026-07-14

**Authors:** Ligia Rodina, Vlad Monescu, Lavinia Georgeta Caplan, Maria Elena Cocuz, Victoria Bîrluțiu

**Affiliations:** 1 Doctoral School of Medicine, Faculty of Medicine, Lucian Blaga University of Sibiu, 550169 Sibiu, Romania; victoria.birlutiu@ulbsibiu.ro; 2 Department of Infectious Diseases, Faculty of Medicine, Transilvania University of Brașov, 500036 Brașov, Romania; maria.cocuz@unitbv.ro; 3 Faculty of Mathematics and Computer Science, Transilvania University of Brașov, 500091 Brașov, Romania; monescu@unitbv.ro; 4 Clinical Hospital of Pneumophthisiology and Infectious Diseases of Brașov, 500118 Brașov, Romania; lavinia.caplan@scpfbibv.ro; 5 Department of Clinical Medicine II, County Emergency Clinical Hospital, Lucian Blaga University of Sibiu, 550169 Sibiu, Romania

**Keywords:** COVID-19, SARS-CoV-2, subacute thyroiditis, autoimmune thyroiditis, thyroid ultrasound, post-COVID condition

## Abstract

*Background and Objectives*: Thyroid dysfunction has been increasingly reported during and after SARS-CoV-2 infection, yet prospective longitudinal studies integrating biochemical and imaging assessments remain limited. The aim of this study was to evaluate the evolution of thyroid function and structural changes during a 12-month follow-up after COVID-19 hospitalization. *Materials and Methods*: This prospective single-center cohort study included adult patients hospitalized with laboratory-confirmed COVID-19 between December 2022 and December 2024. Thyroid-stimulating hormone (TSH), free thyroxine (FT4), free triiodothyronine (FT3), anti-thyroid peroxidase (anti-TPO), and anti-thyroglobulin antibodies (anti-TG) were assessed during hospitalization and at 1, 3, 6, and 12 months. Color Doppler thyroid ultrasound was performed at baseline and 6 and 12 months. Acute disease severity, vaccination status, and corticosteroid exposure were recorded. *Results:* Of the 71 enrolled patients, 67 completed the 12-month follow-up. Subacute thyroiditis occurred in 4/67 patients (6.0%; 95% CI: 1.7–12.6%). After excluding individuals with pre-existing disease, newly detected autoimmune thyroiditis was identified in 8/61 patients (13.1%; 95% CI: 5.8–24.2%). Most cases were mild and self-limited. Ultrasound patterns suggestive of autoimmune thyroiditis increased over time, even in patients with minimal biochemical abnormalities. Thyroid hormone trajectories showed interindividual variability, with most values remaining within reference ranges. Associations with inflammatory markers, disease severity, and vaccination status were weak. *Conclusions*: A subset of patients developed biochemical and structural thyroid changes during the 12 months following COVID-19 hospitalization. These findings highlight the value of targeted thyroid follow-up after COVID-19, particularly in patients with persistent symptoms, borderline TSH values, or ultrasound changes suggestive of autoimmune thyroiditis.

## 1. Introduction

Since the onset of the COVID-19 pandemic, it has become clear that SARS-CoV-2 infection may involve multiple organ systems beyond the respiratory tract, including the endocrine system. The thyroid gland has been repeatedly reported as a potential site of involvement, with biochemical and clinical abnormalities described both during the acute phase of infection and in the post-infectious period [1,2,3,4].

During acute illness, alterations in thyroid function are often attributed to non-thyroidal illness syndrome (NTIS), a common adaptive response to severe systemic disease. However, in addition to NTIS, distinct forms of primary thyroid involvement have been described in association with SARS-CoV-2 infection, including subacute thyroiditis (SAT) and autoimmune thyroid diseases (AITDs), such as Hashimoto’s thyroiditis and Graves’ disease, reported as new-onset conditions or as exacerbations of pre-existing disease [4,5,6,7].

Several mechanisms have been proposed to explain these observations. SARS-CoV-2 may directly affect thyroid tissue, as the gland expresses angiotensin-converting enzyme 2 (ACE2) and transmembrane protease serine 2 (TMPRSS2), which facilitate viral entry into host cells [8,9]. In addition, systemic inflammation during COVID-19, characterized by elevated cytokines such as interleukin-6 (IL-6) and tumor necrosis factor-α (TNF-α), may influence the hypothalamic–pituitary–thyroid axis and peripheral thyroid hormone metabolism, contributing to transient biochemical abnormalities [10,11]. Finally, post-infectious immune dysregulation has been suggested as a potential contributor to autoimmune thyroid manifestations through mechanisms such as molecular mimicry or bystander activation [12,13].

Despite increasing interest in post-COVID endocrine sequelae, several uncertainties remain. Many published studies are retrospective, cross-sectional, or limited to short follow-up periods, making it difficult to characterize the longitudinal trajectory of thyroid abnormalities after infection [4,11,14]. Reported incidence rates vary widely, partly due to differences in patient selection, disease severity, and screening intensity. Moreover, the relative contribution of acute disease severity, systemic inflammation, corticosteroid therapy, and vaccination status to subsequent thyroid alterations remains incompletely defined.

In this context, we conducted a prospective, single-center cohort study to descriptively evaluate thyroid function, thyroid autoimmunity, and ultrasound features in adult patients hospitalized for COVID-19, with follow-up extending to 12 months after infection. The primary objective was to characterize the longitudinal evolution of biochemical and structural thyroid changes and to explore their temporal patterns in relation to acute disease characteristics. Given the prospective design and structured follow-up, the study aimed to provide a detailed longitudinal characterization of thyroid alterations after COVID-19 hospitalization. The novelty of our study lies in the integrated longitudinal assessment of thyroid hormones, autoantibodies, and serial Doppler ultrasound findings in a predominantly mild-to-moderate COVID-19 cohort.

## 2. Materials and Methods

### 2.1. Study Design and Setting

This prospective, single-center observational cohort study was conducted at the Clinical Hospital of Pneumophthisiology and Infectious Diseases in Brașov, a regional referral center for the management of COVID-19 patients in central Romania. Patient recruitment took place during the late pandemic period (2022–2024). Each participant was scheduled for longitudinal follow-up extending up to two years after the acute SARS-CoV-2 infection; however, the present analysis reports findings from the first 12 months. The final sample size reflects the decline in COVID-19 hospitalizations during the later pandemic period, which limited the number of eligible patients.

### 2.2. Participants

Adult patients aged between 18 and 80 years who were hospitalized with laboratory-confirmed SARS-CoV-2 infection, established by reverse transcription polymerase chain reaction (RT-PCR) or high-sensitivity antigen testing, were eligible for inclusion. All participants provided written informed consent and confirmed their availability for scheduled follow-up visits throughout the 12-month observation period.

Patients were excluded if they had a history of thyroid disease requiring long-term thyroid hormone replacement therapy or antithyroid treatment; were receiving chronic interferon, amiodarone, or other medications known to significantly interfere with thyroid function; were on chronic corticosteroid or anticoagulant therapy prior to the COVID-19 episode; or were unable to comply with the planned follow-up schedule. Individuals with previously diagnosed euthyroid autoimmune thyroiditis not requiring treatment were included in the overall longitudinal assessment. However, analyses addressing newly detected autoimmune thyroiditis during follow-up were restricted to participants without known prior autoimmune thyroid disease.

### 2.3. Data Collection

For each participant, demographic characteristics, relevant comorbidities, thyroid-related medical history, SARS-CoV-2 vaccination status, acute COVID-19 severity, and treatments administered during hospitalization were extracted from electronic medical records. Disease severity was categorized according to clinical presentation and oxygen requirements during admission. Information on SARS-CoV-2 vaccination status, including vaccine type and number of administered doses, was obtained from electronic medical records. Laboratory parameters obtained during hospitalization and follow-up visits included inflammatory markers (C-reactive protein, erythrocyte sedimentation rate, ferritin, fibrinogen, and interleukin-6), coagulation parameters (D-dimer), hepatic enzymes (AST, ALT, LDH), and renal function tests. These variables were collected as part of routine clinical assessment and used for descriptive and exploratory analyses. A complete list of recorded variables is presented in Table 1.

### 2.4. Thyroid Function and Imaging Assessment

Thyroid function was evaluated at baseline (during hospitalization) and at 1, 3, 6, and 12 months after SARS-CoV-2 infection. Laboratory assessment included serum thyroid-stimulating hormone (TSH), free thyroxine (FT4), free triiodothyronine (FT3), anti-thyroid peroxidase antibodies (anti-TPO), and anti-thyroglobulin antibodies (anti-TG). Color Doppler thyroid ultrasound was performed at baseline and systematically repeated at 6 and 12 months. Additional ultrasound examinations were conducted when clinically indicated, particularly in the presence of new symptoms suggestive of thyroid involvement or significant biochemical abnormalities. All ultrasound evaluations were performed using high-resolution equipment by a single experienced physician. Ultrasound findings were assessed according to echogenicity, parenchymal homogeneity, and vascularity parameters. Because of the prospective clinical design, the examiner was not formally blinded to clinical information or previous ultrasound findings. Formal intra-observer reproducibility was not assessed.

### 2.5. Follow-Up Protocol

Patients were followed according to a predefined schedule at 1, 3, 6, and 12 months after SARS-CoV-2 infection. At each visit, a comprehensive clinical and endocrine evaluation was performed, including repeat thyroid function tests, thyroid autoantibody measurements, and inflammatory markers. Color Doppler thyroid ultrasound was systematically performed at 6 and 12 months. In addition to these scheduled assessments, interim clinical, biochemical, and ultrasound evaluations were conducted whenever patients developed symptoms suggestive of thyroid involvement, such as anterior cervical pain, palpitations, or unexplained fatigue. This approach allowed for the timely diagnosis of subacute thyroiditis and other thyroid abnormalities that may have occurred between predefined study visits.

### 2.6. Operational Definitions

Subacute thyroiditis (SAT) was defined by the presence of anterior cervical pain or tenderness, elevated inflammatory markers (CRP and/or ESR), and biochemical thyrotoxicosis characterized by suppressed TSH with elevated FT4. A subsequent transient hypothyroid phase followed by recovery was considered supportive of the diagnosis [15,16,17]. Compatible ultrasound findings included focal or diffuse hypoechoic areas with reduced vascularity [15]. Autoimmune thyroiditis (AIT) was defined by characteristic ultrasound findings suggestive of autoimmune thyroiditis, with or without elevated anti-thyroid antibodies, after exclusion of previously known thyroid disease [18]. Non-thyroidal illness syndrome (NTIS) was defined as transient low FT3 levels during the acute phase of illness, with normal or low TSH, in the absence of persistent biochemical or structural thyroid abnormalities at follow-up [19].

### 2.7. Thyroid Laboratory Assessment

Serum thyroid-stimulating hormone (TSH), free thyroxine (FT4), free triiodothyronine (FT3), anti-thyroid peroxidase antibodies (anti-TPO), and anti-thyroglobulin antibodies (anti-TG) were measured using a chemiluminescent immunoassay (CLIA) on a fully automated immunoassay analyzer (MAGLUMI X3, Snibe Diagnostic, Shenzhen, China). The reference intervals were 0.4–4.5 mIU/L for TSH, 8.9–17.2 pmol/L for FT4, 2.6–6.0 pmol/L for FT3, anti-TPO < 10 IU/mL, and anti-TG < 95 IU/mL. All analyses were performed in the same certified laboratory under standardized internal and external quality control procedures.

### 2.8. Study Objectives

The primary objective of this study was to characterize longitudinal changes in thyroid function and ultrasound features during the first 12 months following hospitalization for SARS-CoV-2 infection. Secondary objectives were to describe the occurrence of subacute and autoimmune thyroiditis during follow-up and to explore potential associations between thyroid parameters, inflammatory markers, acute disease severity, and vaccination status. All analyses were conducted within the context of the cohort size and observational study design.

### 2.9. Statistical Analysis

Continuous variables are expressed as mean ± standard deviation or median (interquartile range), depending on data distribution, which was assessed using appropriate normality tests. Comparisons between groups were performed using Student’s *t*-test or the Mann–Whitney U test, as appropriate. Categorical variables were compared using the chi-square test or Fisher’s exact test. Correlations between thyroid parameters and inflammatory markers were evaluated using Spearman’s rank correlation coefficient. Exact 95% confidence intervals were calculated for key proportions. Longitudinal changes in thyroid parameters were primarily assessed descriptively using repeated measurements over time. Given the observational design and sample size, analyses were considered exploratory. All statistical tests were two-sided, and a *p*-value < 0.05 was considered statistically significant. Statistical analyses were performed using IBM SPSS Statistics for Windows, Version 29.0 (IBM Corp., Armonk, NY, USA) and R version 4.4.1 (R Foundation for Statistical Computing, Vienna, Austria).

### 2.10. Ethical Considerations

The study was approved by the Ethics Committee of the Brașov Clinical Hospital for Pulmonology and Infectious Diseases and by the Ethics Committee of “Lucian Blaga” University of Sibiu. All participants provided written informed consent prior to enrollment. The study was conducted in accordance with the Declaration of Helsinki and applicable data protection regulations.

## 3. Results

### 3.1. Cohort Characteristics

**Figure 1:**
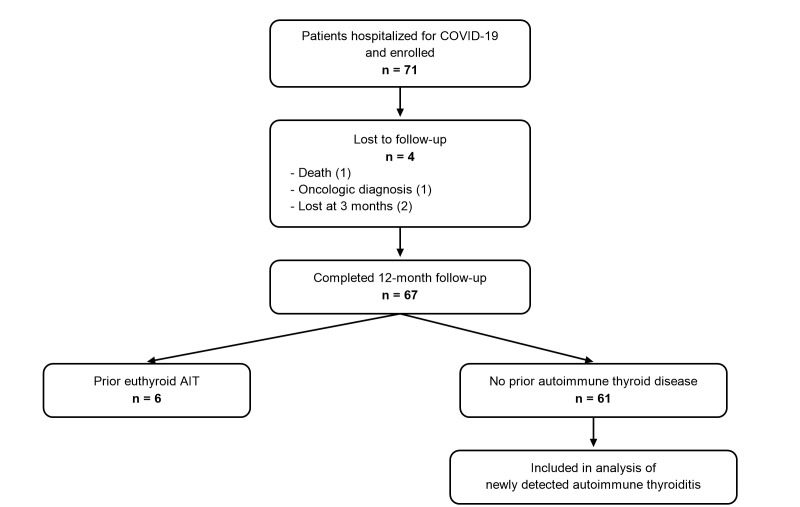
Flowchart of participant inclusion and follow-up. A total of 71 patients hospitalized for COVID-19 were enrolled in the prospective cohort. Four participants were lost to follow-up, resulting in a final cohort of 67 patients who completed the 12-month evaluation. Of these, six had previously known euthyroid autoimmune thyroiditis at baseline and were included in the overall longitudinal assessment but excluded from the analysis of newly detected autoimmune thyroiditis, which was restricted to the remaining 61 participants without known prior autoimmune thyroid disease.

The flow of participants through the study is summarized in Figure 1. A total of 71 patients were enrolled during hospitalization for COVID-19 between December 2022 and December 2024. Four participants were lost to follow-up, including one accidental death, one newly diagnosed oncologic condition, and two patients lost after the 3-month visit, resulting in a final cohort of 67 patients who completed the 12-month evaluation.

Of the 67 patients included in the final analysis, 6 (9.0%) had previously known euthyroid autoimmune thyroiditis at baseline. These individuals were included in the overall longitudinal assessment but were excluded from the analysis of newly detected autoimmune thyroiditis, which was restricted to the remaining 61 participants without known prior autoimmune thyroid disease.

The median age of the study population was 54 years (range 25–78 years), and 54 participants (80.6%) were female. Thirty-six patients (53.7%) had received at least one dose of a SARS-CoV-2 vaccine prior to infection, while 31 (46.3%) were unvaccinated. The majority of vaccinated individuals received the BNT162b2 vaccine (Pfizer Inc., New York, NY, USA; BioNTech SE, Mainz, Germany). Specifically, 25 patients (69.4% of vaccinated individuals) received two doses, 6 (16.7%) received three doses, and 2 (5.6%) received four doses. Additionally, 3 patients (8.3%) received the Ad26.COV2.S (Johnson & Johnson, New Brunswick, NJ, USA) vaccine. Baseline demographic characteristics and clinical data are summarized in Table 2.

### 3.2. Severity of Acute COVID-19 and Administered Treatments

All patients were classified according to predefined national and international severity criteria. Most patients had mild disease (53/67, 79.1%), while moderate and severe forms were less frequent (11/67, 16.4% and 3/67, 4.5%, respectively) (Table 3). Interstitial or ground-glass radiologic changes were observed in 34 patients (50.7%), while normal chest imaging was documented in 33 patients (49.3%). Acute respiratory failure requiring supplemental oxygen occurred in 8 patients (11.9%), and no cases required non-invasive or invasive mechanical ventilation. Antiviral therapy was administered in 39 patients (58.2%), most commonly remdesivir (24/67, 35.8%). Systemic corticosteroids were used in 23 patients (34.3%), and anticoagulation therapy in 34 patients (50.7%). Tocilizumab was administered in one patient (1.5%). Detailed treatment data are summarized in Table 4.

### 3.3. Baseline Correlations Between Inflammatory and Biochemical Markers

**Figure 2:**
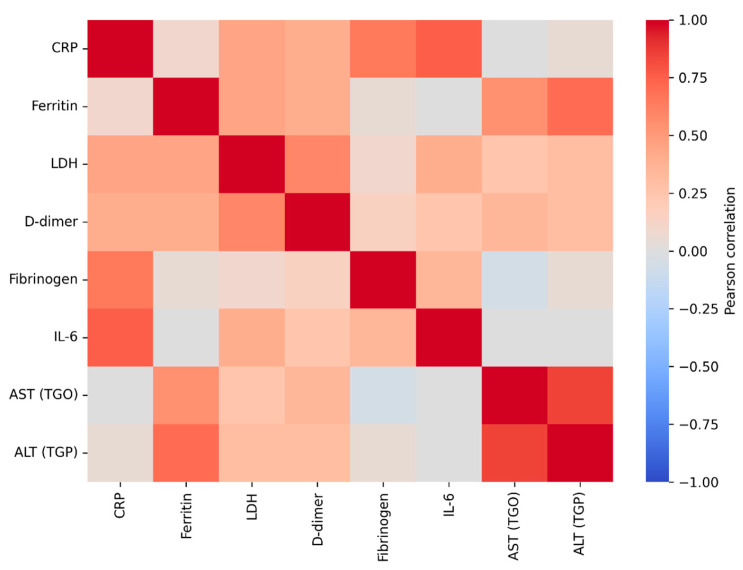
Spearman correlation heatmap of baseline inflammatory and related laboratory markers. The heatmap illustrates correlations between inflammatory markers (CRP, ESR, fibrinogen, IL-6) and biochemical parameters (ferritin, LDH, D-dimer, AST, ALT). Red colors indicate positive correlations, whereas blue colors indicate negative correlations, with color intensity reflecting the magnitude of the correlation coefficient (ρ). Relatively strong positive correlations were observed between hepatic transaminases (AST–ALT), as well as between CRP–IL-6, LDH–D-dimer, and ferritin–ALT, patterns compatible with a coordinated inflammatory response at baseline.

At baseline, several significant correlations were observed among inflammatory, hepatic, and coagulation-related parameters. AST and ALT showed a strong positive correlation (r = 0.894, *p* < 0.001). Ferritin was positively correlated with ALT (r = 0.739, *p* < 0.001) and AST (r = 0.598, *p* < 0.001). LDH was positively correlated with D-dimer levels (r = 0.639, *p* < 0.001). Inflammatory markers including ESR, CRP, and fibrinogen were moderately correlated (r ranging from 0.654 to 0.671, all *p* < 0.001). These correlations are illustrated in Figure 2.

### 3.4. Longitudinal Evolution of Thyroid Hormones

**Figure 3:**
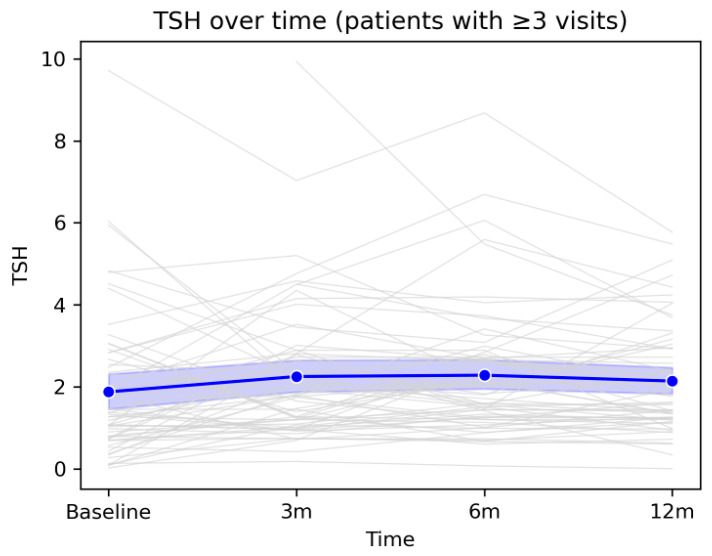
Longitudinal evolution of TSH levels over the 12-month follow-up period after COVID-19 infection. Grey lines represent individual patient trajectories in subjects with at least three study visits, while the blue line depicts the cohort mean with the corresponding 95% confidence interval. A moderate degree of interindividual variability is observed, alongside an overall tendency toward stabilization of TSH values by the end of the follow-up period.

**Figure 4:**
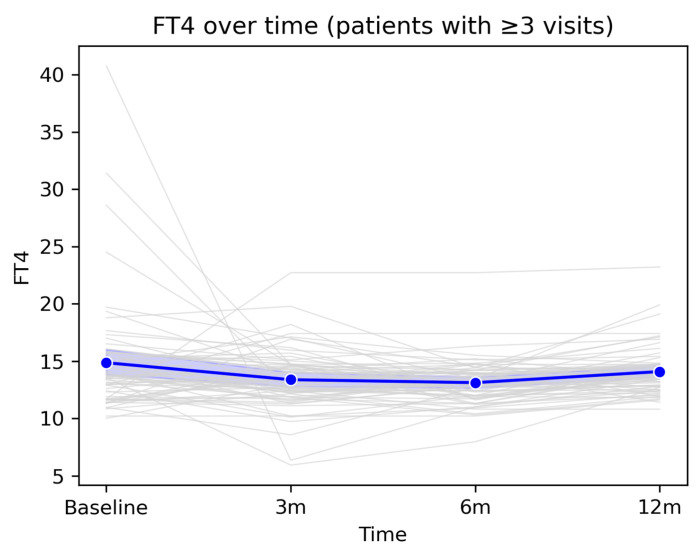
Longitudinal evolution of FT4 levels over the 12-month follow-up period after COVID-19 infection. Grey lines represent individual patient trajectories in subjects with at least three follow-up visits, highlighting interindividual variability in FT4 levels. The blue line indicates the cohort mean at each assessment time point (baseline, 3 months, 6 months, and 12 months), with the corresponding 95% confidence interval displayed as a semi-transparent blue band.

**Figure 5:**
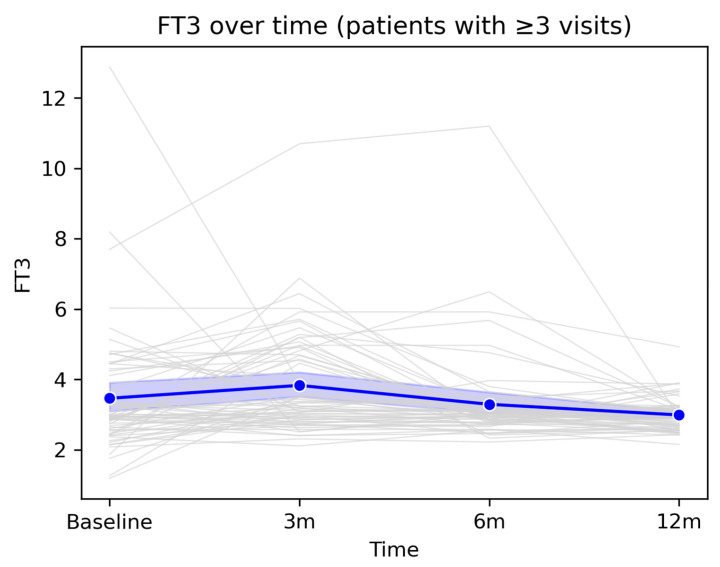
Longitudinal evolution of FT3 levels over the 12-month follow-up period after COVID-19 infection. Grey lines represent individual patient trajectories in subjects with at least three follow-up visits, highlighting interindividual variability in FT3 levels. The blue line indicates the cohort mean at each assessment time point (baseline, 3 months, 6 months, and 12 months), with the corresponding 95% confidence interval displayed as a semi-transparent blue band.

Longitudinal assessment of thyroid function over 12 months demonstrated modest fluctuations in TSH, FT4, and FT3 levels, with substantial interindividual variability. Mean TSH values showed a slight increase at 3 and 6 months, followed by a decrease toward baseline at 12 months. Group-level TSH values remained within the reference range throughout follow-up (Figure 3). FT4 levels demonstrated a mild decline at 3 months, followed by relative stabilization and partial return toward baseline at 12 months (Figure 4). Mean FT4 values remained within the reference interval. FT3 levels showed minor variations over time, with mean values consistently within the physiological range (Figure 5). At the cohort level, no progressive deterioration of thyroid function was observed. For graphical clarity, longitudinal plots include data from baseline and 3, 6, and 12 months.

### 3.5. Occurrence and Patterns of TSH Abnormalities

Among the 67 patients who completed the 12-month follow-up, 23 (34.3%) had at least one TSH measurement outside the reference range during the study period, while 44 patients (65.7%) maintained TSH values within the reference range at all time points. TSH abnormalities were variable and predominantly transient, with both suppressed and elevated values observed during follow-up. In most cases, these alterations did not persist across consecutive visits. Patients with at least one abnormal TSH value were older compared to those with consistently normal TSH levels (*p* = 0.043). Higher TSH values at early follow-up visits (including the 1-month and 3-month assessments) were associated with subsequent TSH abnormalities.

### 3.6. Longitudinal Thyroid Ultrasound Findings

Longitudinal thyroid ultrasound evaluation demonstrated changes in structural thyroid patterns over the 12-month follow-up period. The proportion of patients with a normal thyroid ultrasound appearance decreased from 20 (29.9%) at baseline to 18 (26.9%) at 6 months and 12 (17.9%) at 12 months. Of the 14 participants with ultrasound findings suggestive of autoimmune thyroiditis at 12 months, six had pre-existing euthyroid autoimmune thyroiditis documented at baseline, while eight represented newly detected cases. The presence of thyroid cysts remained stable throughout follow-up, being observed in 24 patients at all evaluation time points. Similarly, thyroid nodules showed minimal variation, being identified in 17 patients at baseline and at 6 months, and in 16 patients at 12 months, without relevant changes in size or ultrasound characteristics.

**Figure 6:**
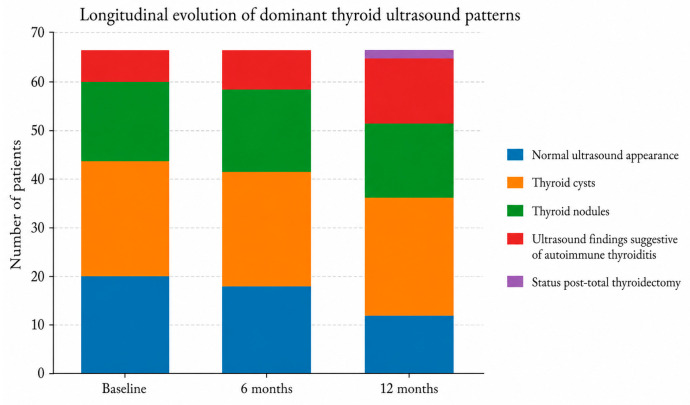
Longitudinal evolution of dominant thyroid ultrasound patterns during the 12-month follow-up after SARS-CoV-2 infection. Stacked bar chart illustrating the distribution of dominant thyroid ultrasound findings at baseline, 6 months, and 12 months. Each participant was assigned to a single dominant ultrasound category at each scheduled assessment. Note: Four cases of subacute thyroiditis were diagnosed during interim clinically indicated evaluations approximately 30–45 days after SARS-CoV-2 infection and are not represented as a separate category in the scheduled ultrasound assessments.

Ultrasound patterns suggestive of autoimmune thyroiditis increased over time, from 6 patients (9.0%) at baseline to 8 (11.9%) at 6 months and 14 (20.9%) at 12 months. Four cases of subacute thyroiditis were diagnosed during interim clinically indicated evaluations performed approximately 30–45 days after SARS-CoV-2 infection. By the scheduled 6-month ultrasound assessment, active sonographic features of subacute thyroiditis had resolved, and these patients were classified according to their contemporaneous ultrasound findings. One patient underwent total thyroidectomy following the diagnosis of papillary thyroid carcinoma during follow-up. The distribution of thyroid ultrasound findings across time points is presented in Table 5 and Figure 6.

### 3.7. Occurrence of Subacute and Autoimmune Thyroiditis During Follow-Up

Subacute thyroiditis (SAT) was identified in 4 patients (6.0%; 95% CI: 1.7–12.6%) during the follow-up period. Clinical symptoms, including anterior cervical pain and systemic inflammatory manifestations, developed approximately 30–45 days after the acute SARS-CoV-2 infection. These cases were diagnosed during interim clinical and ultrasound evaluations performed approximately 30–45 days after SARS-CoV-2 infection in symptomatic patients or in those with suggestive biochemical abnormalities. Because these interim assessments were not systematically performed in the entire cohort, they were not considered a separate predefined follow-up time point within the study protocol. By the 6-month visit, most patients were already in the recovery phase, and no active ultrasound features of SAT were observed at the 12-month evaluation. Detailed hormonal and serological characteristics of patients diagnosed with subacute thyroiditis are presented in Supplementary Figures S1–S4.

Newly detected autoimmune thyroiditis was identified in 8 patients during follow-up. Excluding the 6 participants with previously known euthyroid autoimmune thyroiditis at baseline, this corresponded to 8/61 patients (13.1%; 95% CI: 5.8–24.2%). In all cases, baseline thyroid ultrasound was normal, and structural abnormalities developed during follow-up, most frequently becoming apparent at the 6-month evaluation. Ultrasound findings were characterized by diffuse hypoechogenicity and parenchymal heterogeneity.

Thyroid autoantibody levels showed considerable interindividual variability during follow-up. Some patients developed elevated anti-TPO levels, and others developed elevated anti-TG levels, while several patients showed only low-level or borderline antibody responses despite characteristic ultrasound findings suggestive of autoimmune thyroiditis. Although antibody levels declined over time in some individuals, characteristic ultrasound abnormalities persisted throughout follow-up, suggesting that structural thyroid changes may outlast serological activity. Detailed clinical, serological, and ultrasound characteristics of these patients are presented in Supplementary Table S1. Individual longitudinal trajectories of TSH, anti-TPO, and anti-TG levels are shown in Supplementary Figures S5–S7.

No cases of overt Graves’ disease were identified during the follow-up period.

No consistent associations were observed between SARS-CoV-2 vaccination status and thyroid function abnormalities, thyroid autoantibody positivity, or ultrasound findings during follow-up. The distribution of thyroid alterations was similar between vaccinated and unvaccinated individuals; however, the limited sample size precludes definitive conclusions.

Overall, the longitudinal assessment revealed that thyroid involvement after SARS-CoV-2 infection was generally mild and self-limited. Most hormonal alterations were transient and remained within reference ranges at the cohort level. Subacute thyroiditis occurred infrequently but presented with characteristic clinical and ultrasonographic features and resolved during follow-up. Notably, the proportion of patients with ultrasound patterns suggestive of autoimmune thyroiditis increased over time. In several cases, ultrasound abnormalities persisted despite declining antibody titers and minimal thyroid function abnormalities, indicating that structural thyroid changes may remain detectable after partial serological normalization.

## 4. Discussion

### 4.1. Principal Findings

In this prospective, single-center study with 12-month follow-up after SARS-CoV-2 infection, thyroid-related biochemical and structural changes were observed in a subset of patients, despite the predominance of mild and moderate acute COVID-19. The main findings were the identification of subacute thyroiditis in 4 patients (6.0%) and features consistent with autoimmune thyroiditis in 8 patients during follow-up. Excluding the 6 participants with previously known euthyroid autoimmune thyroiditis at baseline, this corresponded to newly detected autoimmune thyroiditis in 8/61 (13.1%) patients. Most of these abnormalities were mild, heterogeneous, and frequently transient, and progressive thyroid dysfunction was not observed at the cohort level.

An important finding of the present study is that thyroid alterations were documented even in patients without severe acute disease. Most participants did not require advanced respiratory support, and the overall clinical course of thyroid involvement was generally favorable. Cases of subacute thyroiditis were treated with corticosteroids and showed a rapid clinical response, with complete resolution during follow-up, while autoimmune thyroiditis was more often associated with mild or subclinical functional changes rather than overt thyroid failure. These observations are in line with previous reports describing a broad spectrum of post-COVID thyroid manifestations, ranging from transient hormonal abnormalities to inflammatory and autoimmune thyroid disorders [22,23,24,25]. The occurrence of subacute thyroiditis in our cohort is consistent with the growing literature describing this entity after SARS-CoV-2 infection [13,22,26,27]. The temporal profile observed in our patients, with symptom onset approximately 4–6 weeks after acute infection, is also consistent with previously reported post-viral patterns and supports a clinically meaningful temporal association [28,29,30]. At the same time, the favorable evolution observed in all SAT cases, without persistent thyroid dysfunction at 12 months, is in agreement with the generally reversible course reported in other prospective and observational studies [13,22,31].

Autoimmune thyroiditis was identified during follow-up in a smaller but clinically relevant subgroup of patients. In several cases, thyroid autoantibodies were detected during follow-up together with ultrasound changes suggestive of autoimmune thyroiditis. Although antibody titers declined over time in some patients, characteristic ultrasound abnormalities persisted throughout follow-up [18]. Although causality cannot be established in the absence of pre-infection serological data and a non-COVID comparator group, the temporal emergence of these findings after SARS-CoV-2 infection is compatible with a possible role of post-infectious immune dysregulation in susceptible individuals [12,23,32,33]. The persistence of thyroid autoantibody positivity at 12 months in a subset of patients further supports the need for continued follow-up in selected cases. The longitudinal hormone data also provide an important clinical perspective. Although individual trajectories of TSH, FT4, and FT3 were variable, mean values remained within reference ranges throughout follow-up, and no progressive deterioration was observed at the cohort level. This suggests that, in many patients, post-infectious thyroid abnormalities may be limited in magnitude and may evolve without clinically significant long-term dysfunction within the first year. At the same time, the fact that more than one-third of participants had at least one abnormal TSH value during follow-up indicates that thyroid function may remain dynamic during convalescence, particularly when assessed systematically.

Another clinically relevant aspect is the contribution of longitudinal thyroid ultrasound. In addition to biochemical monitoring, serial imaging allowed the identification of structural patterns suggestive of autoimmune thyroiditis that were not always accompanied by marked hormonal abnormalities. This finding suggests that biochemical and structural thyroid changes may not evolve in parallel in all patients and highlights the added value of imaging in selected clinical contexts.

Within the limitations of the study design, these findings provide longitudinal clinical, biochemical, immunological, and ultrasound data on thyroid involvement after COVID-19 and support the value of targeted endocrine follow-up in selected patients.

### 4.2. Subacute Thyroiditis After COVID-19: Clinical and Paraclinical Features

In our prospective cohort, subacute thyroiditis (SAT) was identified in 4 patients (6.0%) during the 12-month follow-up after SARS-CoV-2 infection. This proportion falls within the range reported in previous studies, although direct comparisons remain limited by differences in study design, patient populations, and the intensity of endocrine follow-up [22,29,30,31,34]. Prospective studies with systematic monitoring have generally reported higher detection rates, suggesting that milder or atypical cases may be underrecognized in routine clinical practice.

The temporal pattern observed in our cohort, with symptom onset approximately 30–45 days after the acute infection, is consistent with previously described post-viral latency intervals for subacute thyroiditis, including both SARS-CoV-2-associated cases and those related to other viral infections [28,29,30]. By the scheduled 6-month ultrasound assessment, active sonographic features of SAT had resolved. This time course is compatible with a post-infectious inflammatory mechanism, although causality cannot be established within the present study design.

All cases exhibited clinical, biochemical, and ultrasound features consistent with the classical presentation of SAT, including biochemical thyrotoxicosis, elevated inflammatory markers, and characteristic hypoechoic areas with reduced vascularity on thyroid ultrasound [13,30,31]. Thyroid autoantibodies remained within reference ranges in all patients, supporting the non-autoimmune nature of the condition [28,31]. All patients responded rapidly to corticosteroid therapy, with resolution of symptoms and normalization of thyroid function during follow-up. In some cases, a transient hypothyroid phase preceded recovery, consistent with the typical triphasic evolution of SAT. No patients required long-term thyroid hormone replacement, and the overall clinical course was favorable, in agreement with previous reports [13,22,31].

### 4.3. Autoimmune Thyroiditis After COVID-19

Newly detected autoimmune thyroiditis was identified in 8 of 61 patients (13.1%) without previously known autoimmune thyroid disease during the 12-month follow-up period. In these cases, thyroid ultrasound abnormalities developed during follow-up and were characterized by diffuse hypoechogenicity and parenchymal heterogeneity, consistent with patterns typically described in autoimmune thyroiditis [18]. Thyroid autoantibody levels showed considerable interindividual variability during follow-up. Some patients developed elevated anti-TPO levels, and others developed elevated anti-TG levels, while several patients showed only low-level or borderline antibody responses despite characteristic ultrasound findings suggestive of autoimmune thyroiditis. Detailed clinical, serological, and ultrasound characteristics of these patients are presented in Supplementary Table S1.

In several patients, thyroid autoantibodies were detected during follow-up in the absence of previously known thyroid disease, suggesting a temporal association between SARS-CoV-2 infection and the emergence of autoimmune thyroid features. However, in the absence of pre-infection serological data and a non-COVID comparator group, it cannot be determined whether these findings reflect de novo autoimmune activation or the unmasking of a previously subclinical condition [35,36,37,38].

From a functional perspective, most patients remained euthyroid or developed only mild TSH elevations consistent with subclinical hypothyroidism. No patients required initiation of thyroid-specific treatment during the observation period, and no cases of overt hypothyroidism were documented within the first year of follow-up. These findings suggest that, in this cohort, autoimmune thyroid involvement was generally mild in terms of functional impact over the study period. An important observation is the persistence of thyroid autoantibodies at 12 months in a subset of patients. Although the clinical significance of this finding remains uncertain, data from the general population indicate that the presence of anti-TPO antibodies may be associated with an increased long-term risk of thyroid functional decline [39,40,41]. This highlights the potential relevance of continued follow-up in selected patients with persistent immunological abnormalities.

Our findings are consistent with previous prospective and observational studies reporting the emergence or fluctuation of thyroid autoantibodies following SARS-CoV-2 infection, including in patients without known pre-existing thyroid disease [25,32,42]. However, the available literature remains heterogeneous, and the long-term evolution of post-COVID thyroid autoimmunity has not yet been fully characterized.

Overall, the temporal relationship between SARS-CoV-2 infection and the subsequent detection of autoimmune thyroid features observed in this study is compatible with a possible role of immune-mediated mechanisms in susceptible individuals, although causality cannot be established within the present study design [12,32,33,43].

### 4.4. Thyroid Hormone Dynamics and Determinants of Post-COVID Dysfunction

The longitudinal analysis of thyroid hormone dynamics revealed a heterogeneous but predominantly self-limited post-infectious endocrine response. Despite substantial interindividual variability, mean TSH, FT4, and FT3 values remained within reference ranges throughout the 12-month follow-up and showed a general trend toward stabilization over time. These findings are consistent with previous longitudinal studies suggesting that post-COVID thyroid dysfunction is generally mild and reversible [24,25,32,44].

During the early months after infection, mild fluctuations in TSH were frequently observed, occasionally accompanied by transient reductions in FT3 and less pronounced variations in FT4. In a subset of patients, this pattern was compatible with non-thyroidal illness syndrome (NTIS) or a prolonged NTIS-like profile, reflecting an adaptive response of the hypothalamic–pituitary–thyroid axis to systemic stress rather than primary thyroid disease [19,45,46]. Importantly, these early hormonal abnormalities did not consistently predict persistent thyroid dysfunction, highlighting the importance of longitudinal follow-up when interpreting isolated biochemical alterations.

No consistent associations were identified between thyroid hormone dynamics and inflammatory markers, disease severity, or vaccination status. Thyroid abnormalities were also observed in patients with non-severe acute disease, suggesting that post-COVID thyroid involvement is not restricted to severe infections [25,32,40]. Overall, the findings support a dynamic but largely adaptive endocrine response characterized by mild, reversible alterations and limited evidence of sustained thyroid dysfunction during the first year after infection. However, these exploratory analyses were likely underpowered to detect subtle associations and should therefore be interpreted with caution.

### 4.5. Thyroid Ultrasound Findings and Subclinical Structural Changes

A key finding of the present study is the dissociation between structural thyroid changes and biochemical parameters over time. Longitudinal thyroid ultrasound revealed a progressive increase in patterns suggestive of autoimmune thyroiditis, even in patients with minimal or absent hormonal abnormalities. This observation indicates that structural thyroid involvement may evolve independently of overt biochemical dysfunction and may remain subclinical when assessed solely by serum markers [11,13,44].

At baseline, most patients exhibited either normal ultrasound findings or common benign abnormalities, such as cysts and non-suspicious nodules. These findings remained largely stable throughout follow-up, suggesting that they likely represent incidental background features rather than direct consequences of SARS-CoV-2 infection. In contrast, diffuse hypoechoic and heterogeneous patterns increased over time, supporting the emergence or progression of autoimmune-related structural changes during the post-infectious period [18,40,47].

Subacute thyroiditis was diagnosed during interim clinically indicated evaluations approximately 30–45 days after SARS-CoV-2 infection. By the scheduled 6-month ultrasound assessment, active sonographic features of subacute thyroiditis had resolved, confirming the transient nature and favorable evolution of this condition [13,22,26,31,48]. In patients with newly detected autoimmune thyroiditis, ultrasound abnormalities persisted in several cases despite declining antibody titers, suggesting that structural thyroid changes may outlast serological activity [18,40].

From a clinical perspective, these findings suggest that thyroid ultrasound may provide additional diagnostic information beyond thyroid function testing alone, particularly in patients with persistent symptoms or subtle biochemical abnormalities [18,44,49]. Structural thyroid alterations persisted in a subset of patients despite minimal functional impairment, highlighting the complementary role of imaging and biochemical assessment in the evaluation of post-COVID thyroid involvement. However, the long-term clinical significance of these ultrasound abnormalities remains uncertain [18,37,40].

### 4.6. Clinical Implications

The findings of the present study have potential clinical implications for the evaluation of patients during the post-COVID period, particularly within the broader context of post-COVID condition, where persistent endocrine manifestations have increasingly been recognized [50,51]. Although most thyroid abnormalities were mild and transient, the identification of both functional and structural changes in a subset of patients suggests that thyroid involvement may represent an underrecognized component of post-COVID recovery in selected individuals [42,52]. Thyroid function assessment may be particularly relevant in patients presenting with persistent or non-specific symptoms, such as fatigue, palpitations, or unexplained weight changes [42]. In addition, individuals with borderline TSH values or early post-infectious hormonal fluctuations may represent a subgroup at higher risk of subsequent thyroid dysfunction [18,44,49].

Our findings also suggest that early post-infectious thyroid function may have prognostic relevance, as initial TSH values were associated with subsequent thyroid abnormalities. However, given the observational design and limited sample size, these observations should be interpreted with caution and require confirmation in larger studies. Thyroid ultrasound may provide additional diagnostic value, particularly in patients with persistent biochemical abnormalities or suspected autoimmune involvement, as structural changes may occur in the absence of overt hormonal dysfunction.

Overall, these results support a targeted, individualized approach to thyroid evaluation after SARS-CoV-2 infection, rather than systematic screening [42,52]. Further prospective studies are needed to define optimal follow-up strategies and to identify patients at risk of persistent thyroid dysfunction.

### 4.7. Strengths and Limitations

The present study has several strengths that enhance its relevance within the current literature on post-COVID-19 thyroid involvement. First, its prospective design allowed for systematic and standardized follow-up over a 12-month period after SARS-CoV-2 infection, reducing the risk of selection and recall bias commonly associated with retrospective studies. The predefined assessment time points enabled accurate characterization of the temporal dynamics of thyroid function.

The extended follow-up duration represents an additional strength, providing a longitudinal perspective that remains relatively limited in post-COVID research [42]. This approach allowed differentiation between transient post-infectious hormonal alterations and more persistent or evolving thyroid dysfunction, including the identification of newly detected autoimmune thyroiditis during follow-up.

A further strength is the integrated evaluation of thyroid involvement, combining hormonal parameters (TSH, FT4, FT3), immunological markers (anti-TPO and anti-TG antibodies), and systematic thyroid ultrasound assessment. This multimodal approach improved the detection of subclinical or structural abnormalities and allowed a more comprehensive characterization of post-COVID thyroid changes. In addition, the single-center design, with uniform inclusion criteria and consistent follow-up protocols, enhanced internal consistency.

However, several limitations should be considered. The relatively small sample size limits statistical power, particularly for exploratory analyses, and precludes robust multivariable modeling. No formal a priori sample size calculation was performed, and the resulting incidence estimates are associated with relatively wide confidence intervals, which may limit the precision of the reported frequencies. The single-center design may also limit generalizability to other populations and healthcare settings. Furthermore, all participants were recruited among hospitalized COVID-19 patients, which may have resulted in the selection of individuals with more symptomatic disease and limits the generalizability of the findings to non-hospitalized post-COVID-19 populations. The predominance of female participants (80.6%) may also have influenced the observed frequency of autoimmune thyroid abnormalities and may limit the generalizability of the findings.

The absence of a non-COVID control group restricts the ability to determine whether the observed incidence of subacute thyroiditis and autoimmune thyroiditis differs from that expected in the general population. Therefore, the observed thyroid abnormalities cannot be definitively attributed to SARS-CoV-2 infection, and the findings should be interpreted as temporal associations rather than evidence of causality.

Pre-infection thyroid function, thyroid autoantibody status, and thyroid ultrasound findings were unavailable. Consequently, it cannot be conclusively determined whether cases classified as newly detected autoimmune thyroiditis represent de novo post-infectious autoimmune activation or the unmasking of previously unrecognized subclinical thyroid disease.

A small proportion of patients had pre-existing subclinical autoimmune thyroiditis at baseline, and although their number was limited, a potential influence on longitudinal outcomes cannot be entirely excluded. In addition, unmeasured factors such as stress, seasonal variation, or treatments during the acute phase may have influenced thyroid function.

Despite these limitations, the prospective design, standardized follow-up, and integrated biochemical, immunological, and imaging assessment provide valuable longitudinal insight into thyroid alterations after SARS-CoV-2 infection and support the need for further investigation in larger, multicenter cohorts.

### 4.8. Future Directions

Further research is needed to better characterize the medium- and long-term impacts of SARS-CoV-2 infection on thyroid function and structure [53]. Large, multicenter prospective studies are required to validate these findings and improve generalizability across diverse populations. In addition, studies integrating more detailed immunological assessments may help clarify the mechanisms underlying post-COVID thyroid dysfunction and distinguish transient inflammatory changes from evolving autoimmunity. Longer follow-up periods are also necessary to determine whether persistent thyroid autoantibodies are associated with an increased risk of overt thyroid disease over time.

## 5. Conclusions

This prospective 12-month follow-up study showed that biochemical and structural thyroid changes can be identified in a subset of patients after SARS-CoV-2 infection, including patients with predominantly mild or moderate acute disease. Thyroid hormone abnormalities were mostly mild and transient, with an overall tendency toward normalization over time despite heterogeneous individual trajectories. A smaller subgroup developed subacute thyroiditis during follow-up, while newly detected autoimmune thyroiditis was identified in 8/61 (13.1%) participants without known prior autoimmune thyroid disease. Subacute thyroiditis was uncommon but clinically and ultrasonographically well characterized, whereas ultrasound patterns suggestive of autoimmune thyroiditis increased over time, sometimes in the absence of overt biochemical dysfunction. Early post-infectious TSH values may be associated with subsequent thyroid dynamics. Overall, these findings are compatible with a possible association between SARS-CoV-2 infection and transient inflammatory or autoimmune thyroid alterations in susceptible individuals. Given the observational design, larger prospective studies are needed to confirm these associations and better define their long-term clinical implications.

## Figures and Tables

**Table 1:** Variables Collected in the Study.

Variable Category	Included Parameters
Demographic data	Age, sex
Comorbidities	Diabetes mellitus, arterial hypertension, chronic kidney disease, cardiovascular diseases, active oncological diagnosis, COPD/bronchial asthma
COVID-19 severity	Clinical form (mild, moderate, severe), oxygen requirement, acute respiratory failure, radiological findings (chest X-ray/pulmonary CT)
Acute-phase treatment	Antivirals (Remdesivir, Favipiravir, Nirmatrelvir/ritonavir, Molnupiravir), corticosteroids (Dexamethasone, Methylprednisolone, etc.), anticoagulants, Tocilizumab
Inflammatory markers	ESR, CRP, fibrinogen, ferritin, IL-6
Thyroid function	TSH, FT4, FT3
Thyroid autoantibodies	anti-TPO, anti-TG
Imaging investigations	Color Doppler thyroid ultrasound
Additional factors	SARS-CoV-2 vaccination status

**Table 2:** Baseline demographic characteristics, COVID-19 vaccination status, and history of autoimmune thyroid disease in the final study cohort (*n* = 67).

Characteristic	*n* (%)
Sex	
Female	54 (80.6)
Male	13 (19.4)
SARS-CoV-2 vaccination status	
Vaccinated	36 (53.7)
Unvaccinated	31 (46.3)
Type of vaccine (among vaccinated)	
Pfizer—2 doses	25 (69.4)
Pfizer—3 doses	6 (16.7)
Pfizer—4 doses	2 (5.6)
Johnson & Johnson	3 (8.3)
History of autoimmune thyroid disease	
Prior autoimmune thyroid disease	6 (9.0)
No prior autoimmune thyroid disease	61 (91.0)

Legend: Data are presented as number (percentage). Percentages for vaccine type are calculated among vaccinated participants.

**Table 3:** Clinical Severity and Respiratory/Radiologic Features in the Study Cohort.

Severity Category	n (%)
Mild disease	53 (79.1)
Moderate disease	11 (16.4)
Severe disease	3 (4.5)
Interstitial/ground-glass changes	34 (50.7)
Normal imaging	33 (49.3)
Acute respiratory failure	8 (11.9)

Legend: Clinical severity categories were assigned according to national and international COVID-19 severity criteria [20,21]. Data are presented as number (percentage).

**Table 4:** Treatments administered during the acute COVID-19 phase (*n* = 67).

Treatment	*n* (%)
Antiviral therapy (any)	39 (58.2)
Remdesivir	24 (35.8)
Favipiravir	7 (10.4)
Nirmatrelvir/ritonavir	6 (9.0)
Molnupiravir	2 (3.0)
Systemic corticosteroids	23 (34.3)
Anticoagulation (prophylactic or therapeutic)	34 (50.7)
Tocilizumab	1 (1.5)

Legend: Data are presented as number (percentage). Patients receiving antiviral therapy may have received one of the listed agents. Anticoagulation includes both prophylactic and therapeutic regimens.

**Table 5:** Longitudinal evolution of dominant thyroid ultrasound patterns during the 12-month follow-up after SARS-CoV-2 infection.

Ultrasound Pattern	Baseline (*n* = 67)	6 Months (*n* = 67)	12 Months (*n* = 67)
Normal ultrasound appearance	20 (29.9%)	18 (26.9%)	12 (17.9%)
Thyroid cysts	24 (35.8%)	24 (35.8%)	24 (35.8%)
Thyroid nodules	17 (25.4%)	17 (25.4%)	16 (23.9%)
Ultrasound findings suggestive of autoimmune thyroiditis	6 (9.0%)	8 (11.9%)	14 (20.9%)
Status post-total thyroidectomy	0 (0.0%)	0 (0.0%)	1 (1.5%)

Note: Although multiple ultrasound features could coexist in the same patient, for the purposes of longitudinal analysis, each individual was assigned to a single dominant ultrasound category at each scheduled evaluation time point, defined as the finding with the greatest clinical relevance. Consequently, the sum of categories equals the total number of patients evaluated at each time point.

## Data Availability

The data presented in this study are available on reasonable request from the corresponding author. The data are not publicly available due to privacy and ethical restrictions.

## Supplementary Materials

The following supporting information can be downloaded at: https://www.mdpi.com/article/10.3390/medicina62071354/s1. Table S1. Clinical, biochemical, serological, and ultrasound characteristics of patients with newly detected autoimmune thyroiditis during follow-up. Figure S1. TSH levels in patients with subacute thyroiditis identified during follow-up. Figure S2. FT4 levels in patients with subacute thyroiditis identified during follow-up. Figure S3. FT3 levels in patients with subacute thyroiditis identified during follow-up. Figure S4. Thyroid autoantibody levels in patients with subacute thyroiditis identified during follow-up. Figure S5. Longitudinal evolution of TSH values in patients with newly detected autoimmune thyroiditis. Figure S6. Longitudinal evolution of anti-TPO antibody levels in patients with newly detected autoimmune thyroiditis. Figure S7. Longitudinal evolution of anti-Tg antibody levels in patients with newly detected autoimmune thyroiditis.

## Abbreviations

ACE2	angiotensin-converting enzyme 2
AITD	autoimmune thyroid disease
Anti-TG	anti-thyroglobulin antibodies
Anti-TPO	anti-thyroid peroxidase antibodies
COVID-19	coronavirus disease 2019
CRP	C-reactive protein
ESR	erythrocyte sedimentation rate
FT3	free triiodothyronine
FT4	free thyroxine
IL-6	interleukin 6
LDH	lactate dehydrogenase
NTIS	non-thyroidal illness syndrome
RT-PCR	reverse transcription polymerase chain reaction
SARS-CoV-2	severe acute respiratory syndrome coronavirus 2
SAT	subacute thyroiditis
TSH	thyroid-stimulating hormone
US	ultrasound
