# Supplementary material for: Post-COVID-19 Thyroid Dysfunction and Autoimmune Ultrasound Changes: A 12-Month Prospective Cohort Study
Source: Medicina (Kaunas). 2026 Jul 14;62(7):1354. doi: 10.3390/medicina62071354 (PMC13413736; doi:10.3390/medicina62071354)
Supplement: Supplementary file 1 [file medicina-62-01354-s001.zip › medicina-4364485-supplementary.pdf]

**Supplementary Table S1. Clinical, biochemical, serological, and ultrasound characteristics of patients with newly detected autoimmune thyroiditis during follow-up.**

| Patient | Maximum anti-TPO level (IU/mL) | Maximum anti-TG level (IU/mL) | Thyroid function during follow-up | Ultrasound findings | Time of first detection |
|---|---|---|---|---|---|
| AIT-1 | 1000 | 693 | Euthyroid | Diffuse hypoechogenicity and heterogeneity | 6 months |
| AIT-2 | 6.52 | 26.1 | Euthyroid | Diffuse hypoechogenicity and heterogeneity | 6 months |
| AIT-3 | >1000 | 1943 | Mild TSH elevation | Diffuse hypoechogenicity and heterogeneity | 6 months |
| AIT-4 | 588 | 61 | Mild TSH elevation | Diffuse hypoechogenicity and heterogeneity | 6 months |
| AIT-5 | 921 | 77.6 | Euthyroid | Diffuse hypoechogenicity and heterogeneity | 12 months |
| AIT-6 | 919 | 78.5 | Euthyroid | Diffuse hypoechogenicity and heterogeneity | 12 months |
| AIT-7 | 3.43 | 1538 | Mild TSH elevation | Diffuse hypoechogenicity and heterogeneity | 12 months |
| AIT-8 | 0.984 | 120 | Euthyroid | Diffuse hypoechogenicity and heterogeneity | 12 months |

Reference values: anti-TPO <10 IU/mL; anti-TG <95 IU/mL. All patients classified as newly detected autoimmune thyroiditis demonstrated ultrasound findings consistent with autoimmune thyroiditis. Thyroid autoantibody levels showed marked interindividual variability during follow-up. Thyroid dysfunction was generally mild and limited to isolated or transient TSH elevations.

**Supplementary Figure S1.** TSH levels in patients with subacute thyroiditis identified during follow-up.

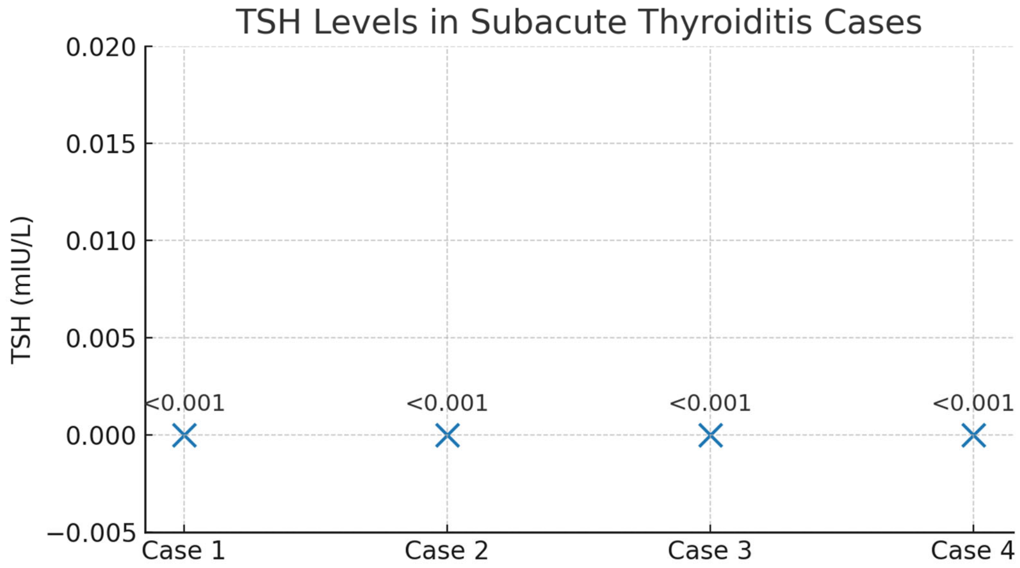


Legend: Individual TSH values measured at the time of subacute thyroiditis diagnosis in the four affected patients. All cases exhibited profound TSH suppression below the assay detection limit (<0.001 mIU/L), consistent with the thyrotoxic phase of subacute thyroiditis.

**Supplementary Figure S2.** FT4 levels in patients with subacute thyroiditis identified during follow-up.

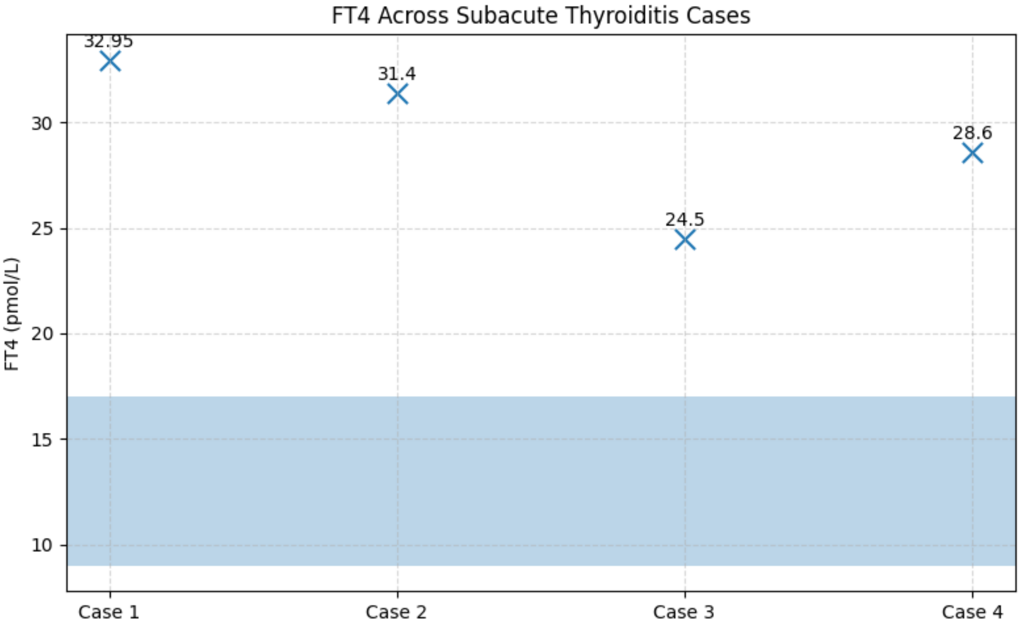


Legend: Individual serum FT4 concentrations measured at the time of subacute thyroiditis diagnosis in the four affected patients. The shaded area represents the reference range for FT4 (8.9–17.2 pmol/L). All cases exhibited markedly elevated FT4 levels, consistent with the thyrotoxic phase of subacute thyroiditis.

**Supplementary Figure S3.** FT3 levels in patients with subacute thyroiditis identified during follow-up.

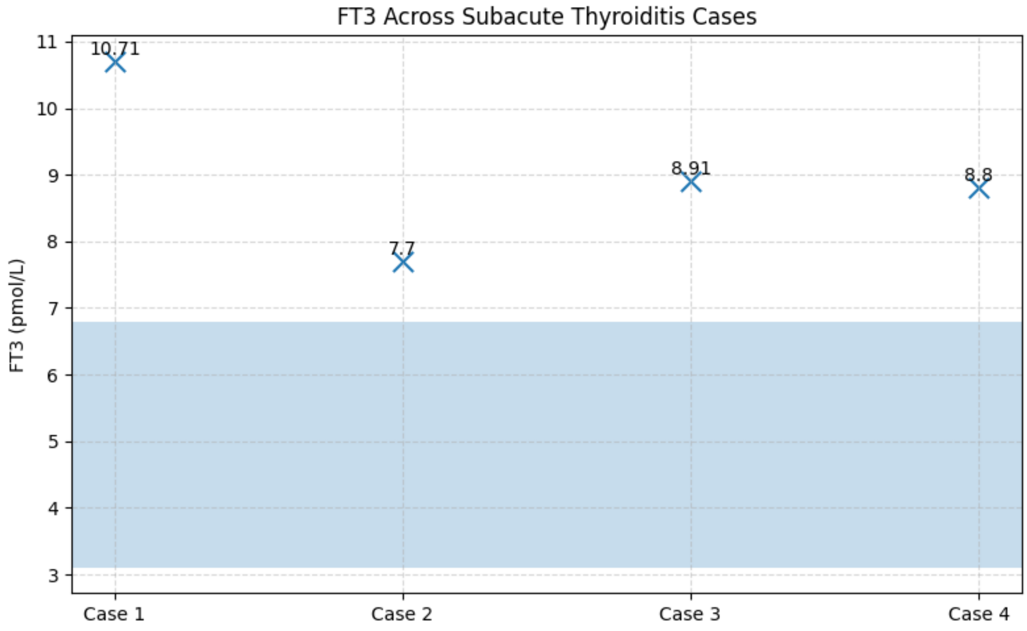


Legend: Individual serum FT3 concentrations measured at the time of subacute thyroiditis diagnosis in the four affected patients. The shaded area represents the reference range for FT3 (2.6–6.0 pmol/L). All cases exhibited elevated FT3 levels, consistent with the thyrotoxic phase of subacute thyroiditis.

**Supplementary Figure S4.** Thyroid autoantibody levels in patients with subacute thyroiditis identified during follow-up.

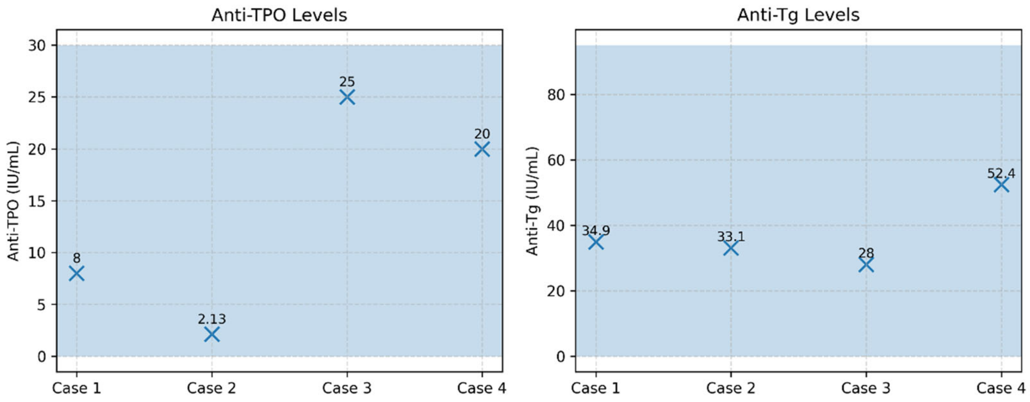


Legend: Individual serum anti–thyroid peroxidase (anti-TPO) and anti–thyroglobulin (anti-Tg) concentrations measured at the time of subacute thyroiditis diagnosis in the four affected patients. The shaded areas represent the respective reference ranges (anti-TPO < 10 IU/mL; anti-thyroglobulin < 95 IU/mL). All values remained within normal limits, supporting the non-autoimmune nature of subacute thyroiditis.

**Supplementary Figure S5.** Longitudinal evolution of TSH values in patients with newly detected autoimmune thyroiditis.

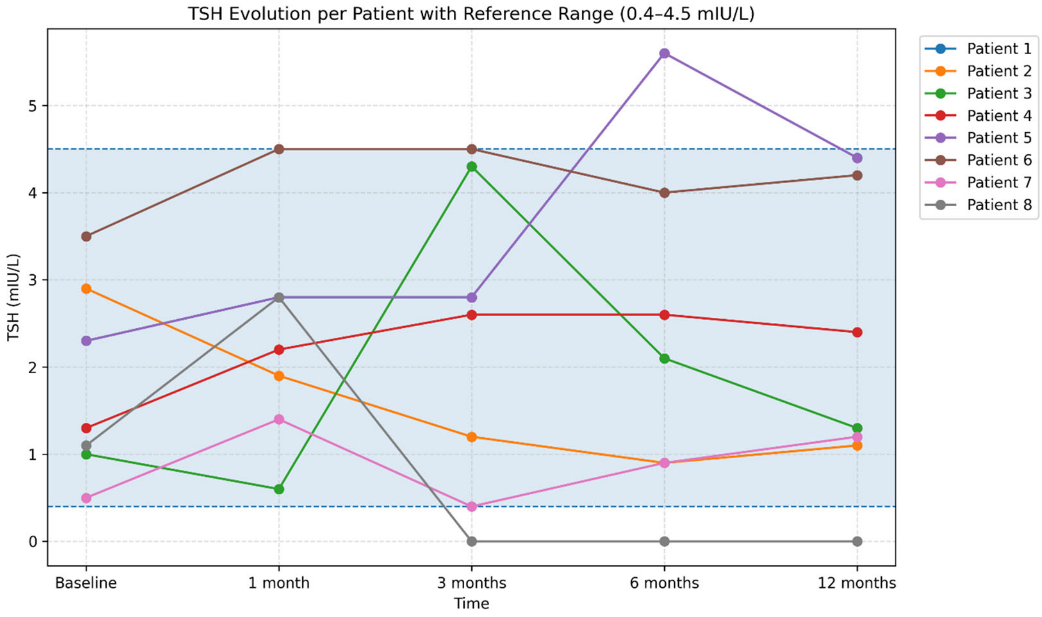


Legend: Each line represents the longitudinal TSH trajectory of an individual patient diagnosed with autoimmune thyroiditis. The shaded area indicates the reference range for TSH (0.4–4.5 mIU/L), allowing visualization of values within and outside normal limits over the follow-up period

**Supplementary Figure S6.** Longitudinal evolution of anti-TPO antibody levels in patients with newly detected autoimmune thyroiditis.

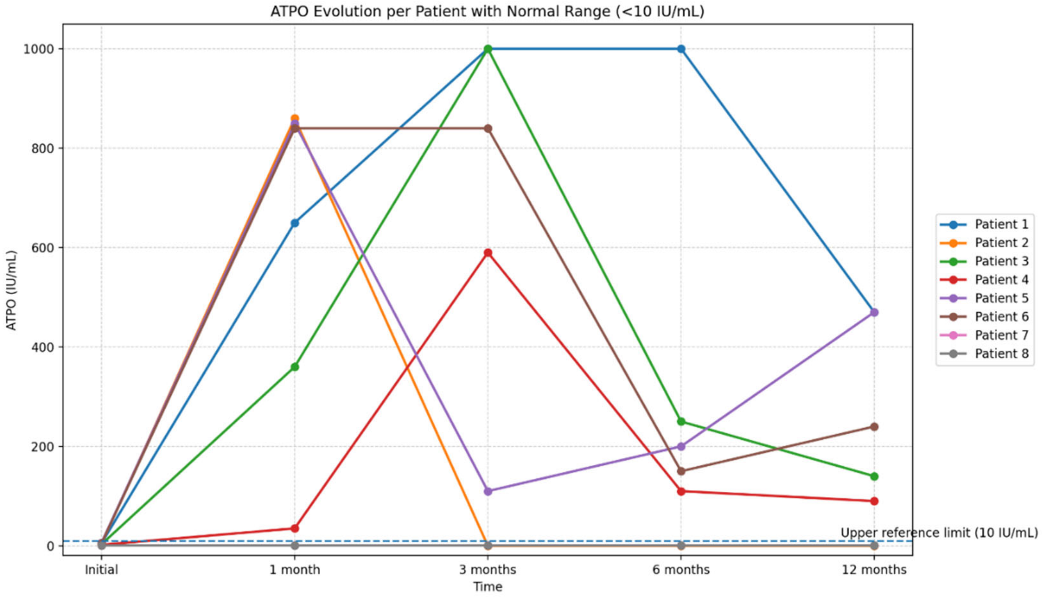


Legend: Each line represents the longitudinal trajectory of anti–thyroid peroxidase (anti-TPO) antibody levels in an individual patient diagnosed with autoimmune thyroiditis. The dashed horizontal line indicates the upper limit of the normal reference range (10 IU/mL), allowing visualization of antibody elevations and their evolution over time.

**Supplementary Figure S7.** Longitudinal evolution of anti-Tg antibody levels in patients with newly detected autoimmune thyroiditis.

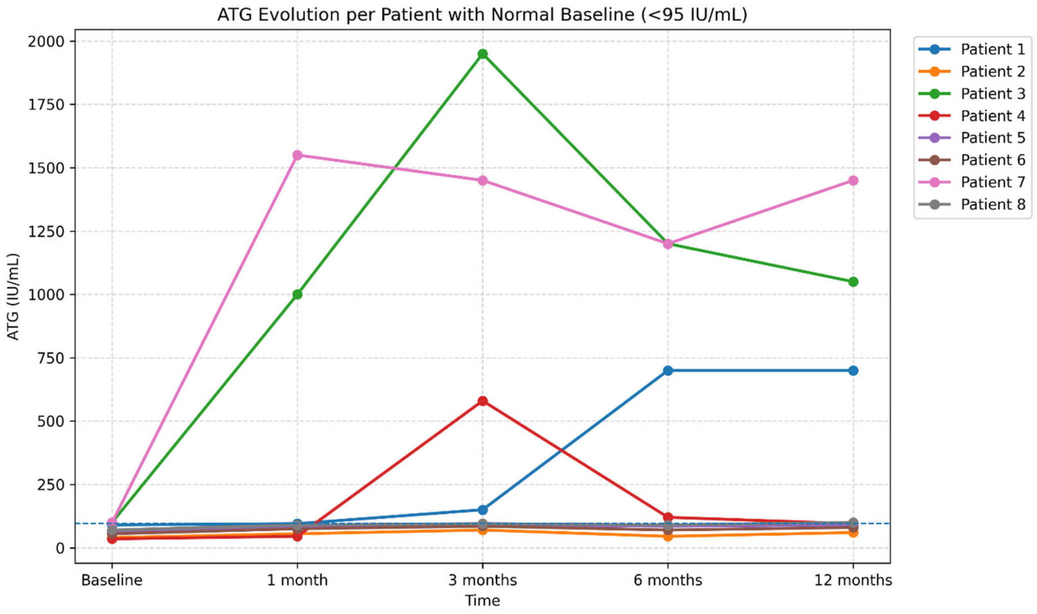


Legend: Each line represents the longitudinal trajectory of anti–thyroglobulin (anti-Tg) antibody levels in an individual patient diagnosed with autoimmune thyroiditis. The dashed horizontal line indicates the upper limit of the normal reference range (95 IU/mL), allowing visualization of antibody elevations and their evolution over time.
